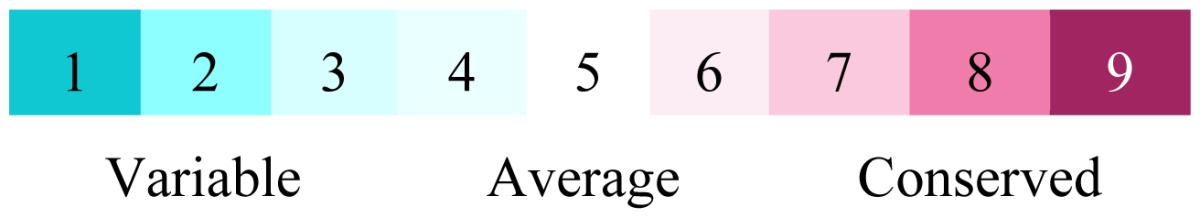# Plant High-Affinity Potassium (HKT) Transporters Involved in Salinity Tolerance: Structural Insights to Probe Differences in Ion Selectivity

**DOI:** 10.3390/ijms14047660

**Published:** 2013-04-09

**Authors:** Shane Waters, Matthew Gilliham, Maria Hrmova

**Affiliations:** 1 Australian Centre for Plant Functional Genomics and Waite Research Institute, University of Adelaide, Glen Osmond, South Australia 5064, Australia; E-Mail: shane.waters@acpfg.com.au; 2 School of Agriculture, Food and Wine, Waite Research Institute and ARC Centre of Excellence in Plant Energy Biology, University of Adelaide, Glen Osmond, South Australia 5064, Australia; E-Mail: matthew.gilliham@adelaide.edu.au

**Keywords:** bacterial TrkH K^+^ transporter, cation exclusion, protein structure and function, selectivity filter and pore, structural analysis

## Abstract

High-affinity Potassium Transporters (HKTs) belong to an important class of integral membrane proteins (IMPs) that facilitate cation transport across the plasma membranes of plant cells. Some members of the HKT protein family have been shown to be critical for salinity tolerance in commercially important crop species, particularly in grains, through exclusion of Na^+^ ions from sensitive shoot tissues in plants. However, given the number of different HKT proteins expressed in plants, it is likely that different members of this protein family perform in a range of functions. Plant breeders and biotechnologists have attempted to manipulate *HKT* gene expression through genetic engineering and more conventional plant breeding methods to improve the salinity tolerance of commercially important crop plants. Successful manipulation of a biological trait is more likely to be effective after a thorough understanding of how the trait, genes and proteins are interconnected at the whole plant level. This article examines the current structural and functional knowledge relating to plant HKTs and how their structural features may explain their transport selectivity. We also highlight specific areas where new knowledge of plant HKT transporters is needed. Our goal is to present how knowledge of the structure of HKT proteins is helpful in understanding their function and how this understanding can be an invaluable experimental tool. As such, we assert that accurate structural information of plant IMPs will greatly inform functional studies and will lead to a deeper understanding of plant nutrition, signalling and stress tolerance, all of which represent factors that can be manipulated to improve agricultural productivity.

## 1. Background

In sequenced genomes, around 30% of all genes are predicted to encode integral membrane proteins (IMPs) [1,2] that are fully embedded in the phospholipid bilayer of a biological membrane. These proteins usually make up around 50% of the total mass of a membrane, but can account for as much as 75% [3]. IMPs are crucial to cell function, as they are a vital point of interaction between cell organelles and the cytoplasm and between the cell as a whole and the extracellular environment. Common types of IMPs include proteins involved in energy transduction (e.g., bacteriorhodopsins, [4]), cell adhesion (e.g., integrins, [5]), catalytic function (e.g., curdlan synthase, [6]), protein-protein interaction (e.g., hyaluronan receptors, [7]) and transport (e.g., aquaporins, [8]). IMPs receive and transmit signals, as well as control the movement of solutes across membranes [9,10]. Those solutes that have high molecular mass or carry charge (e.g., ions, metabolites and sugars), cannot diffuse across phospholipid bilayers, so their movement across a biological membrane is facilitated by transport proteins [9,11]. The transport rate across biological membranes of small and non-polar solutes and gases (e.g., water, urea, ammonia and CO_2_) can also be mediated by IMPs [12]. Thus, solute transporters are known to be the key players of many well-characterised aspects of physiology in mammalian and plant systems [13–15]. A demonstration of the importance of solute transporters and channels is that they are predicted to be targets of more than half of all pharmaceuticals on the market [1,16]. Despite this importance, structural knowledge of IMPs lags behind that of soluble proteins, with less than 400 unique structures of IMP resolved as of December 2012 [16,17]. Notably, fewer than ten of these 400 unique structures are from plants [18].

The scarcity of structural knowledge about IMPs is due to the additional challenges associated with obtaining IMP structures as opposed to determining the structures of soluble proteins [2,16,18]. These include: (i) the difficulty in obtaining sufficient amounts of IMPs from native or recombinant sources; (ii) recovering IMPs from membranes without denaturing them; (iii) frequent cytotoxicity issues when over-expressing IMPs in recombinant systems and (iv) the complex crystallisation conditions of IMPs.

This article focuses on a particular class of IMP; the plant High-affinity Potassium Transporters (HKTs). HKT transporters occur only in plants, but have sequence and functional similarity to the TrkH/KtrB classes of cation transporters from bacteria and fungi [19,20] and are considered to have a similar structure to these proteins [21,22]. HKTs are segregated into two sub-groups based on their transport selectivity. Group 1 are described as Na^+^ uniporters, while group 2 are thought to allow both Na^+^ and K^+^ transport (and possibly symport of these ions under specific conditions) and Na^+^ uniport at high Na^+^ concentrations. Individual plant species often contain multiple *HKT* genes, e.g., rice has nine, although only seven are functional (in the Nipponbare cultivar) [23]. The HKT proteins are of particular interest to plant biologists, as some members of this class have an important role in helping plants to tolerate high soil salinity, which represents a major agricultural problem worldwide [24,25].

## 2. HKT Transporters and Their Function

### 2.1. Function of HKT Transporters Is Important for Plants Tolerating Soil Salinity

Salinity has come to mean the occurrence of salts (primarily sodium chloride, NaCl) in ground water or the soil solution at levels that inhibit the growth of plants [25]. The actual concentration of salts needed to have a negative influence on plant growth varies and is dependent on many factors, including plant species, soil type and water availability [25]. From an agricultural perspective, soil is usually considered saline when the electrical conductivity (ECe) of the soil solution exceeds 4 dS/m [24,25]. Both intra- and inter-specific natural variation in salinity tolerance exists in plants [24,26]. Sensitive species, such as *Arabidopsis thaliana* and *Oryza sativa*, struggle to survive in the presence of 100 mM NaCl, whereas *Atriplex amnicola* (saltbush) can complete its lifecycle in 600 mM NaCl [24]. Intraspecific variation is common and probably best documented in cereals [27,28]. The mechanisms that result in salt tolerance and account for variation within and between species are numerous, but unsurprisingly, solute transport proteins are often critical, particularly those involved in Na^+^ transport [24,29].

HKT proteins have been characterised predominantly as monovalent cation transporters [30], although reports of Mg^2+^ and Ca^2+^ permeability in some members has been proposed sporadically [31,32]. HKT proteins-mediated transport of Na^+^ is known to be an important component of salinity tolerance in several species, including *Arabidopsis*[26,33,34], rice [22,28,35] and wheat [36–38]. *HKT* genes are frequently discovered underlying quantitative trait loci (QTL) that explain significant variation in salinity tolerance within mapping populations of wheat [37,39,40]. It has been demonstrated that a wide range of alleles for specific *HKT* genes can be present and that differences in these alleles may explain a component of the natural variation in salinity tolerance in *Arabidopsis*[26,41]. For example, Baxter and co-workers [26,41] used genome-wide association mapping, genetic complementation and gene expression studies to identify *cis*-regulatory expression level polymorphisms at the AtHKT1;1 locus. Some reports challenge the assumption that Na^+^ exclusion leads to better salinity tolerance [26,27], with either no correlation or higher Na^+^ concentration in the shoot and lower *HKT* expression correlating with better salt tolerance, while many other reports find the opposite [28,37]. Regardless, *HKT* alleles are an important component of salt tolerance conferred by either strategy. This paradox may be explained by simultaneous differences and natural variation in the other components that underpin salinity tolerance, such as the ability to tolerate the effects of salt accumulation in the shoot or cope with the osmotic components of salinity [24].

The important function of HKTs in assisting plants to survive high soil salinity has been confirmed in gene knockout experiments that made plants more sensitive to salt [33,34,42] and through transgenic expression of *HKT* genes in other plant species, *i.e.*, expression of the *AtHKT1;1* gene in rice allowed the rice plants to grow better under saline conditions [35].

While it is clearly very beneficial to manipulate *HKT* gene expression in crop plants to improve their salinity tolerance in some instances, as recently demonstrated by Munns *et al.*[36], it is important to properly understand function, regulation and expression patterns of the HKT transporters to achieve the desired result. For example, Møller *et al.*[43] overexpressed the *Arabidopsis HKT1;1* gene using a constitutive promoter and a promoter that was specific for stele cells of roots. Transgenic plants overexpressing *AtHKT1;1* constitutively accumulated large amounts of salt in the shoot and were relatively more salt sensitive than control plants, whilst plants over-expressing the same gene specifically in root stele cells accumulated less salt in the shoot and became more salt tolerant [43]. Evidence also exists that different HKT proteins may have different roles within a plant, so it is important to choose the correct allele, when modifying HKT expression to improve salt tolerance. TmHKT1;5-A is located in the cells around the root xylem, where the transporter moves Na^+^ down its electrochemical gradient, which in most physiological circumstances is likely to be out of the xylem [36]. Similarly, AtHKT1;1 is thought to be present around xylem and phloem tissues in the root and shoot, and it also increases Na^+^ retention in the root directly [44]. AtHKT1;1 has also been proposed to recirculate Na^+^ in the phloem, although this function has been questioned [24,30,34]. The possible involvement of HKTs in Na^+^ recirculation is still controversial and has been discussed in detail in a recent review by Hauser and Horie [45]. On the other hand, TaHKT2;1, OsHKT2;1 and OsHKT2;4 appear to be expressed in the outer part of the root, including root hairs, and may actually provide an entry point for Na^+^ into plant roots from the soil solution. In these circumstances, downhill electrochemical gradient for Na^+^ entry into plant cells is proposed to assist with high affinity K^+^ entry into roots when K^+^ is at low concentrations in the soil solution [32,46–48]. With this in mind, it is clearly important to have a comprehensive understanding of how a particular HKT protein functions, before attempting to manipulate expression of that *HKT* or the selection of that allele and its promoter to increase the productivity of a plant species or a variety grown in saline soil.

### 2.2. Ion Selectivity of HKT Transporters

The discovery of plant HKT proteins was made by Schachtman and Schroeder [46], who identified a plant transport protein, which could rescue a mutant yeast strain (*Saccharomyces cerevisiae* CY162) defective in K^+^ transport. A cDNA of the wheat-root plasmid encoding the *HKT1* gene permitted mutant yeast growing on media containing 30 μM K^+^[46]. These authors subsequently expressed this *HKT1* gene in *Xenopus laevis* oocytes and showed that the resulting transport protein was selective for the K^+^ ions, while allowing transport of some Cs^+^ and Rb^+^, but only of trace amounts of Na^+^ or NH_4_^+^. For these reasons, this transport protein was subsequently designated as a “High-affinity Potassium Transporter” (HKT) [46]. However, this protein, now known as TaHKT2;1 [49], is now thought to be a Na^+^/K^+^ symporter at micromolar concentrations of Na^+^ and a Na^+^ uniporter at millimolar concentrations of Na^+^, after more detailed analysis in *Xenopus* oocytes [50,51].

There are numerous *HKT* genes in commercially important grain species [46], but there is only one in the model plant *Arabidopsis*, designated *AtHKT1;1*[52,53]. Most functional studies have indicated that HKT proteins are monovalent cation transporters, with the cation selectivity varying between individual members of the HKT family [46,54,55], although some findings have suggested divalent cation transport by HKT proteins [31,32]. Some HKT proteins are highly selective for Na^+^, while others are more promiscuous and will move Na^+^ and K^+^, e.g., OsHKT2;1 and OsHKT2;2, respectively [55]. Further, some HKT proteins even change their selectivity depending on an ionic environment, e.g., EcHKT1;1 [56]. Many individual *HKT* genes have now been cloned and expressed in various heterologous systems, to evaluate and characterise transport selectivity and behaviour of the specific HKT transport protein. These includes *HvHKT2;1* expressed in yeast [57], *AtHKT1;1* in *Xenopus* oocytes [34], yeast [53] and *E. coli*[53], *EcHKT1;1* and *EcHKT1;2* in *Xenopus* oocytes and *E. coli*[56,58] and *TaHKT2;1* expressed in yeast and *Xenopus* oocytes [46,50]. These experiments in heterologous systems describe HvHKT2;1 as a Na^+^ selective uniporter [57] and OsHKT2;1 and TaHKT2;1 as Na^+^/K^+^ symporters at low Na^+^ concentrations and a Na^+^ uniporter at high Na^+^ concentrations [34,46,50,53,59]. EcHKT1;1 and EcHKT1;2 have been reported to be permeable to Ca^2+^ and Mg^2+^, as well as being Na^+^/K^+^ symporters, even at high Na^+^ concentrations [56,58]. Several authors have observed Na^+^/K^+^ symport by the AtHKT1;1, HvHKT1;5, Ni-OsHKT1;5 and Po-OsHKT1;5 proteins in yeast, which has not been observed in *Xenopus* oocytes [46,57,60]. These observations lead to uncertainty, as to what would be the actual behaviour of HKT proteins *in planta*.

This uncertainty is compounded by paucity of direct evidence showing Na^+^/K^+^ symport in wheat [61], barley [62] and rice [63] roots, calling into question the putative role of HKT2-type proteins in plants in the uptake of K^+^, in K^+^ deficient conditions, using the electrochemical gradient for Na^+^[57,64]. This means that either the results from heterologous systems are incorrect or the activity of the HKTs *in planta* has been masked in these particular experiments through the activity of other plant proteins or in the specific conditions imposed. At the same time, there is evidence of HKT activity from plants that mirrors the activity found in oocytes and yeast for instance Na^+^/K^+^ symport in Arabidopsis [65] and for OsHKT2;1 in a plant cell expression system [55]. Speculation as to the accuracy of extrapolating HKT function *in planta* from heterologous systems has been called into question by a number of groups for reasons ranging from artefacts induced by the choice of vector used for protein overexpression to the inadvertent ascribing of endogenous channel activity of heterologous systems to that of HKTs [48,66]. This extends to the observation of divalent cation transport (Mg^2+^ and Ca^2+^) by OsHKT2;4 when expressed in *Xenopus* oocytes [32], which could not be reproduced in a subsequent study [48]. This is something that the researchers need to be mindful of, when interpreting data from various heterologous expression systems [55,66].

While the transport selectivity of some HKT transporters is well established, the actual mechanism of transport is less clear. Whether HKT predominantly mediates Na^+^, K^+^, Mg^2+^ or Ca^2+^, there have been several proposed mechanisms as to how the HKT proteins mediate movement of these ions. It was originally suggested that H^+^ symport may provide the gradient needed to move ions across the plasma membrane [46,67], although subsequent experimental evidence seemed to suggest that this is unlikely. Active transport by HKT proteins powered by the ATP hydrolysis has been ruled out, due to the lack of a recognisable ATP-binding domain [46]. Both *in vitro* and *in planta* experiments now indicate that the most likely mechanism of HKT proteins is to facilitate the movement of monovalent cations down their electrophysiological gradient [24,68]. Some HKTs potentially having Na^+^/K^+^ symport capacity when either the Na^+^ or K^+^ gradient allows the other ion to enter cells against its electrochemical gradient [50,56,59]; however, this mechanism is highly dependent on specific ionic conditions imposed on heterologous systems, which may not be relevant to *in planta* function. Before manipulating HKT-mediated cation movement *in planta* within the context of altering salinity tolerance in plants, it would be beneficial to describe the characteristics of transport mechanisms of monovalent cations by HKT proteins in general. It would also be useful to know what the structural determinants of selectivity are for specific HKT.

Expression patterns and tissue localisation varies significantly between different HKT transporters, likely reflecting *in planta* function of the individual proteins [32,34,44]. There is a consensus that all HKT proteins characterised so far are plasma membrane IMPs [24,32,34,44,45]. Tissue localisation however, varies significantly. OsHKT1;5 [22] and TaHKT1;5 [37] are expressed in the cells around the root xylem in rice and wheat, while OsHKT1;4 [22] and TaHKT1;4 [38] are expressed in the corresponding cells in the leaf sheath. AtHKT1;1, on the other hand, is expressed around the phloem and xylem tissues in *Arabidopsis*[33,34]. TaHKT2;1 and OsHKT2;1 have quite different localisation though, as these proteins appear to be expressed on the exterior of root and root hairs rather than around the vascular tissue [32,46] and assist with K^+^ and Na^+^ entry into roots [69].

## 3. Molecular Structure of Plant HKT Transporters

Despite the importance of HKT transport proteins, there are no resolved three-dimensional (3D) structures of these proteins in the Protein Data Bank [70]. The structural prediction information for the HKT proteins is based mostly on bioinformatical analyses using bacterial homologues, such as the KcsA K^+^ channel [71]. Recently, molecular models of the rice HKT transporters OsHKT1;5 and OsHKT1;4, based on a crystal structure of the bacterial TrkH K^+^ transporter from *Vibrio parahaemolyticus* (VpTrkH) [21], have been constructed, and the structural data were reconciled with transcriptomic and physiological data [22]. A VpTrkH crystal structure was used here, as this protein and the rice HKT transporters share functional properties as a consequence of sequence and structural similarities [21,22].

### 3.1. Molecular Modelling of Plant HKT Transporters

Protein structure determines protein function [18,72,73]. Therefore, the 3D structure of a protein provides vital information as to how a protein functions [73]. Often proteins with very different sequences can have similar structure and function, although the reverse case can occur, where proteins of very similar amino acid sequences have very different functions [60]. For instance, the plant *Equisetum arvense* (horsetail) has very high levels of silicon (Si), but when Gregoire *et al.*[8] searched the plant’s genome using the sequences of known Si transporters, they were unable to find any homologues. When a family of Si transporters were eventually identified in *Equisetum arvense*, the genes were found to be similar to sequences of aquaporin genes, while the predicted structure was similar to the other Si transporters [8]. Often singular amino acid residues play critical roles in structure of proteins. For example, it has been demonstrated that a single amino acid substitution (Pro to Leu) can substantially alter the functional behaviour of a bacterial PilQ secretin, allowing it to auto-assemble into multimeric assemblies *in vitro*, a process that requires association with another protein for the native PilQ secretin [74].

**Figure 1:**
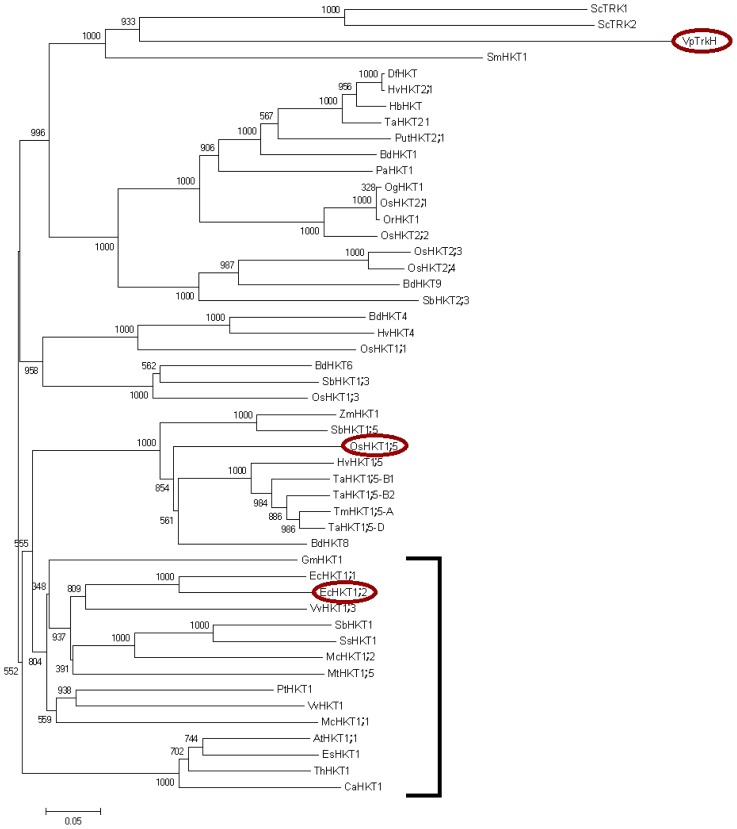
Phylogenetic analysis of plant HKT transporters. All published sequences were retrieved from the NCBI database and aligned with ClustalX2 [79]. The phylogenetic tree was constructed based on aligned protein sequences using Neighbour-Joining algorithm [80] with a Bootstrap value of 1000 using ClustalX2 [79]. The scale bar indicates the substitution rate per site. All dicotyledonous HKT entries (and none of monocotyledonous ones) are bracketed. The red circles denote proteins that are shown in Figure 2.

**Figure 2:**
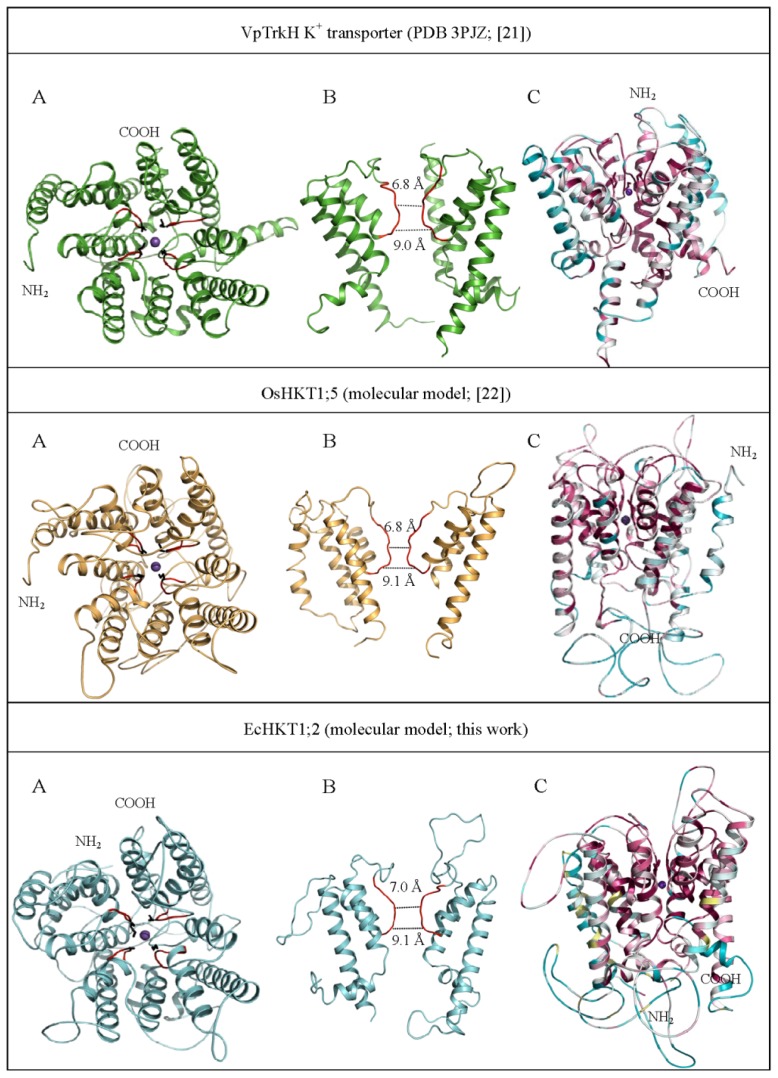
Cartoon representations of 3D structure of VpTrkH (PDB 3PJZ) (top panels) and molecular models of the *Oryza sativa* OsHKT1;5 (middle panels) and *Eucalyptus camaldulensis* EcHKT1;2 (bottom panels) transporters. (**A**) The overall folds of transporters are coloured in green (VpTrkH) orange (OsHKT1;5) and cyan (EcHKT1;2). Purple spheres indicate the K^+^ (VpTrkH) and Na^+^ (OsHKT1;5 and EcHKT1;2) ions. The selectivity filter signatures illustrated in black sticks are: Gly-Gly-Gly-Gly for VpTrkH and Ser-Gly-Gly-Gly for OsHKT1;5 and EcHKT1;2. The residues contained in the selectivity pores are coloured in red. These residues are likely to affect pore rigidity and dispositions of residues controlling cation selectivity and transport rates; (**B**) The cut-out images show geometry of selectivity pores. Their width dimensions are indicated; (**C**) Cartoon representations of VpTrkH, OsHKT1;5 and EcHKT1;2 colour-coded by conservation score of amino acid residues as predicted by ConSurf [81]. The views in panels C are rotated by 90° along the *x*-axis with respect to the views in panels A. The NH_2_- and COOH-termini of the transporters are shown.

Since 2006, when the current naming convention for HKT proteins was proposed [46], there have been a lot of new sequences added to public databases, and therefore, new structural bioinformatics analyses can be performed. To this end, we have performed a phylogenetic analysis of 46 amino acid sequences of known plant HKT proteins from mono- and di-cotyledonous plant species (Table 1) and constructed a phylogenetic tree (Figure 1). Protein sequences were obtained using the BLAST tool from the NCBI database [75] to search for plant sequences that relate to the EcHKT1;2 (AF176036_1), OsHKT1;5 (A2WNZ9.2) and AtHKT1;1 (Q84TI7.1) sequences (Table 1). Many of the annotations available have only been assigned from sequences and are annotated as “Predicted protein, cation transporter HKT1-like” or similar. However, all of the annotated sequences cluster as predicted [49,76], with type 1 and type 2 HKT entries in separate clades. The three proteins standing separate at the top of the tree, *i.e.*, ScTRK1 and ScTRK2 from *Saccharomyces cerevisiae* and VpTrkH from *V. parahaemolyticus* (Figure 1), represent yeast and bacterial K^+^ transporters/symporters that have been included into the analysis. The VpTrkH protein was used as the template structure for molecular modelling shown in Figure 2. The phylogenetic tree (Figure 1) clearly shows that all of the type 2 HKT proteins are in the clade below these three proteins, while the type 1 HKT proteins are in the bottom half of the tree. As predicted by Platten *et al.*[46], there are no dicotyledonous HKT proteins in the type 2 clade. However, it can be noted that the type 1 HKT entries have been subdivided into monocotyledonous and dicotyledonous groups (Figure 1). All the dicotyledonous HKT entries (and none of the monocotyledonous HKT entries) are marked by the bracket at the bottom of the tree. This suggests that a divergence between the monocotyledonous and dicotyledonous *HKT* genes must have occurred early during evolution of both types of plants. It is unclear as yet whether there are any major functional differences between the dicotyledonous and monocotyledonous HKT proteins; however, the eucalyptus (dicotyledonous) EcHKT1;1 and EcHKT1;2 are the only HKT proteins known to be sensitive to solution osmolarity [58]. As noted by Møller and Tester [77], care should be taken, when extrapolating transport selectivity data obtained from dicotyledonous (e.g., *Arabidopsis*) to monocotyledonous species (e.g., rice and wheat). Another recently published phylogenetic analysis [78] suggested segregation between HKTs from simple bryophytes and lycophytes and HKTs from higher plants. That analysis only included *HKT* genes from five species [78], but combined with the phylogenetic analysis included in this study, the possibility is raised that in future, as more information becomes available, HKTs may be reclassified into more subclasses then the current two.

Currently, there is very little experimentally derived structural knowledge about any HKT protein. There are no resolved structures of any HKT protein [70]. However, the topology of the AtHKT1;1 protein was examined by Kato *et al.*[52]. This was determined by inserting various tags (FLAG, PhoA alkaline phosphatase and *N*-glycosylation sites) into the AtHKT1;1 protein sequence and then synthesising the protein in vesicles in a cell-free system or expressing in *E. coli*. The synthesised protein was probed, based on the various tags to determine, on which side of the vesicle/cell membrane the individual tags locate. The data showed that the AtHKT1;1 protein had eight membrane-spanning helices, and the NH_2_- and COOH-termini of AtHKT1;1 were located both on the cytoplasmic side of the membrane, when the protein was expressed in *E. coli*. Various experiments have also been done to mutate individual amino acid residues in order to determine which residues are critical to transport selectivity and function [82,83].

In the absence of experimentally determined structure, it is possible to infer structural data from comparisons with known structures or from computational predictions, such as comparative (homology) modelling [16,72]. These methods are limited by the availability of appropriate templates. For example, as there are no 3D structures of the HKT proteins available, the models of the rice OsHKT1;5 protein and the eucalyptus EcHKT1;2 (unpublished data) could provide this predictive structural information (Figure 2). OsHKT1;5 is a Na^+^ selective transporter [28], EcHKT1;2 is a Na^+^/K^+^ symporter and possibly transports Mg^2+^ and Ca^2+^, as well [56,58]. Both models were built based on the crystal structure of the bacterial K^+^ selective transporter VpTrkH [21], as previously described [22]. Briefly, the sequence of EcHKT1;2 was aligned with that of the VpTrkH K^+^ transporter (Protein Data Bank 3PJZ) [21], and the resultant alignments were tested for the positions of secondary structures. The manually corrected aligned sequences were used as input parameters to generate a molecular model of EcHKT2;1 in complex with Na^+^ using Modeller 9v8 [84]. The best model of EcHKT1;2 was selected from 40 models based on the Modeller 9v8 objective function and the most favourable energy scoring parameters.

The bioinformatics and structural analyses of the plant HKT cation transporters showed that these proteins have the conserved “selectivity filter” motif of Ser-Gly-Gly-Gly for type 1 HKT proteins and Gly-Gly-Gly-Gly for type 2 HKT proteins [49]. Type 1 HKT proteins are highly selective for Na^+^, while type 2 HKT could facilitate movement of Na^+^ and K^+^. Mutating the Ser residue of a type one HKT to Gly would change the transport characteristics of the protein to be similar to the type 2 HKT transporters, at least in the *Xenopus* oocyte expression system [76]. OsHKT2;1 is an exception to this rule, in that the protein is defined as a class 2 HKT, but has the Ser-Gly-Gly-Gly selectivity filter motif [55,76]. EcHKT1;2 is also something of an exception, as this protein has the Ser-Gly-Gly-Gly motif of type 1 HKT, but transports K^+^, as well as Na^+^, which is usually associated with the type 2 HKT proteins [49,56]. This suggests that other structural elements besides the key selectivity filter and pore residues (in K^+^ channels known as the pore helix residues [20]) may help in determining the substrate selectivity of HKT proteins. However, without a resolved 3D structure of HKT proteins, it is challenging to predict which specific motifs of the protein sequence are responsible for transport selectivity. Therefore, at the moment, it is necessary to experimentally determine transport selectivity of each HKT of interest, rather than predict the ion selectivity from its sequence.

Also displayed in Figure 2 (panels C) is the 3D structure of VpTrkH and the molecular models of OsHKT1;5 and EcHKT1;2 that are colour-coded according to the conservation values assigned by the ConSurf Server [81]. This analysis reports conservation of individual amino acid residues in each structure [81]. Cartoon representations of molecular models of OsHKT1;5 and EcHKT1;2, and the crystal structure of VpTrkH indicate that a series of the putative selectivity filter residues occur on the extracellular side of the protein [46,76]. These residues (Figure 2, highlighted in black in panel A) are located in the narrowest point in pores through each of the HKT proteins, which supports the hypothesis that these residues are the primary determinants of substrate selectivity of these transporters. Further, there is a short segment consisting of about seven amino residues (marked red in Figure 2, panels A and B) lining the narrow part of pores before they open up to a cavity. This cavity would not restrict the movement of individual monovalent ions. The conformation of this short loop is likely to be the key determinant of cation permeation into the pore helix of the HKT proteins. While the four selectivity filter residues are critical in this regard (Ser-Gly-Gly-Gly *versus* Gly-Gly-Gly-Gly), the ConSurf analysis showed that most of the remaining amino acid residues forming the pore helices are highly conserved and are, therefore, also likely to be critical for transport function. Last, but not least, the ion conducting channel is bound on the cellular interior by a highly conserved Arg residue, which is a unique feature of the TrkH transporters and almost all bacterial superfamily of K^+^ transporters and has not been observed in any other known K^+^ channel structures [21,85]. Remarkably, these highly conserved Arg and neighbouring Gly residues (Gly-Arg motif) with the highest conservation score of 9 as assigned by ConSurf [81], occur in both plant HKT transporters (data not shown).

Table 2 lists the amino acid residues lining the narrow pore of all three proteins displayed in Figure 2 (coloured in red in panels A and B), that are colour-coded in Table 2 according to conservation score predicted by ConSurf. The pore regions are rich in the Thr and Ser residues, and while both HKT proteins contain an Arg-His pair at the beginning of the third loop of the pore, none of the proteins have any acidic residues prior to the selectivity filter. The presence of polar residues, such as Thr and Ser, in particular, their oxygen atoms along the backbone and side-chain oxygen atoms around the entrance to the pores, is likely to help to co-ordinate cations as they enter the selectivity filter. On the other hand, the Glu residues immediately after the selectivity filter in the HKT proteins present a negative charge at the narrowest point of the selectivity filter, which would prevent any anion permeability, such as, *i.e.*, Cl^−^.

It is surprising that EcHKT1;2 transports K^+^ in *Xenopus* oocytes, while the selectivity filter motif suggests that EcHKT1;2 is a type 1 HKT with Na^+^ selective transport. This is likely to become more apparent if multiple HKT of each clade and of known selectivity are included in the same analysis. However, at this point, it is premature to explain this behavioural discrepancy, as another reason could exist besides the homology modelling prediction based on a K^+^ selective transporter. Future modelling against the structures of Na^+^ selective transporters and Na^+^/K^+^ symporters, when they become available, may reveal the underlying structural reason for an unusual transport behaviour of EcHKT1;2’s. It would also be advisable to confirm the transport behaviour of EcHKT1;2 *in planta* and in other systems, as currently the transport selectivity of EcHKT1;2 has only been defined using yeast and *Xenopus* oocytes, which may not always accurately reflect true function of the EcHKT1;2 protein in its native environment.

### 3.2. Why Is There So Little Structural Data for HKT Transporters?

While IMPs make up a significant proportion of the mass of cell membranes, many membrane proteins are only present on specific membranes, e.g., on a plasma membrane or on a specific organelle [3]. Consequently, individual proteins often occur in very small amounts in their native environments [11]. Limitations in space in the membrane and in co-factors needed for successful membrane insertion can result in overexpressed proteins failing to insert and forming mis-folded “inclusion bodies” in cells [86]. These inclusion bodies can sometimes be solubilised and refolded [86], but it is essential to make sure that refolded proteins are functional before attempting crystallisation, as the structure of a mis-folded and non-functional protein is of little value [18].

Soluble proteins can be expressed with a secretion signal, so the protein is secreted into the growthmedium, where it has limited effect on the survival of the cells. IMPs have to be expressed into a cell membrane to fold correctly and, consequently, may have severe effects on cell survival [31,87], requiring specific media and growth conditions to generate sufficient protein without disturbing the cell culture [11,88].

Purification of IMPs can be performed using standard protein purification methods, such as expressing with a His tag on the protein and using a nickel/cobalt-based affinity chromatography to isolate a protein effectively. However, these methods require the addition of a surfactant to solubilise IMPs. The choice of surfactant is critical, as a surfactant must be efficient enough to separate IMPs from the membrane, without denaturing the protein and must have appropriate chemical structure to protect hydrophobic surfaces of membrane proteins from solution to prevent agglutination, while at the same time, not obscuring the tag being used for purification [89,90].

Crystallisation of IMPs also requires a mixture of hydrophobic and hydrophilic environments [18,90]. This can be achieved by including phospholipids and sometimes gentle surfactants in the crystallisation mixtures [91]. The phospholipids are essential for IMPs to achieve a correct conformation, as they mediate hydrophobic matching that is required between IMP and its native membrane environment [92]. The hydrophilic surfaces of IMPs must be exposed and not obscured by a lipid or surfactant to allow protein-protein interactions, or IMPs do not form an ordered lattice and a crystal [89]. Unfortunately, lipids and surfactants in a protein crystal can interfere with X-ray diffraction; therefore, multiple combinations of additives may have to be tried to find conditions, under which a crystal diffracts well [89].

Das *et al.*[93] have noted that because the new environment, after IMP, is extracted from its native environment and can change protein structure, it is necessary to test the functionality of extracted IMPs. This is usually possible with an enzyme, but may not be practical with transport proteins. However, comparing structures obtained under different conditions, when they exist, can provide an insight into different forms that IMP can take. This is important as crystallised IMP may not be in the exact native conformation. Including substrates or co-factors can increase the likelihood of IMPs taking on a native conformation. It is also important to remember that a crystal structure is a ‘freeze frame’ image of a dynamic molecule, which moves between different states (*i.e.*, open and closed forms of a transporter). Thus, structures with and without an ion can give clues as to the changes that occur, as transport proteins mediate their function, *i.e.*, transport.

## 4. Conclusions and Perspectives

Transport selectivity of some HKT proteins is defined, but there is insufficient data comparing different alleles or mutations to allow a plant breeder to rationally select for a specific allele [37,38,94]. The expression patterns of the HKT proteins *in planta* also need to be more clearly defined. Knowing what cations a particular HKT transporter permeates across a membrane bilayer only reveals a part of the protein function. Exactly which membrane the ions are crossing, on a tissue and a cellular level, will determine the actual effect *in planta*. Expressing marker genes with the HKT promoters and probing for a presence RNA of a specific *HKT* gene (*i.e.*, *in situ* PCR) can reveal the tissues that the protein, or at least its RNA, is expressed in. However, localising the protein ultimately requires a method of detecting the actual protein, not just the corresponding RNA. For example, in the case of HKT proteins, this would undoubtedly require the development of highly specific antibodies [32].

Proteins rarely act in isolation. There are interactions with other proteins or cellular components, which activate, repress and otherwise modulate function of a protein. To date, no data have been reported that identified interaction partners for any HKT protein. This is an area which should be investigated, as it may turn out that the most effective way of modifying HKT function *in planta* is to modify their interaction partners, e.g., to increase the activity of a specific kinase or other modifying enzyme that might regulate transport function.

It would also be highly desirable to determine 3D structures of HKT transport proteins, as homology modelling can only take us so far in understanding the mode of action of HKT proteins at the molecular levels. There are also other experimental methods available to gain insights into protein structure, such as small-angle X-ray scattering and circular dichroism spectroscopy, which reveal the shapes and content of secondary structures within protein folds, respectively.

Last, but not least, performing experiments to analyse transport selectivity in clean systems, without endogenous proteins, *i.e.*, in proteo-liposomes instead of yeast or *Xenopus* oocytes systems, would help to clarify the actual ion selectivity and rates of transport of individual HKT transporters. Finally, it would be worthwhile to perform more detailed mutational analysis of amino acid residues [83] besides those positioned in selectivity filters and pores, especially those that are predicted to be highly conserved across various types of HKT proteins.

## Figures and Tables

**Table 1:** List of entries used for phylogenetic analysis of plant High-affinity Potassium Transporters (HKTs) proteins (*cf*. Figure 1). Accession numbers of protein sequences were obtained with the BLAST tool from the NCBI database to search for sequences that relate to the OsHKT1;5, EcHKT1;2 and AtHKT1;1 entries.

Protein	Species	Common name	Accession number
AtHKT1;1	*Arabidopsis thaliana*	Thale cress	Q84TI7.1
BdHKT1	*Brachypodium distachyon*	Purple false brome	XP_003560515.1
BdHKT4	*Brachypodium distachyon*	Purple false brome	XP_003581628.1
BdHKT6	*Brachypodium distachyon*	Purple false brome	XP_003570995.1
BdHKT8	*Brachypodium distachyon*	Purple false brome	XP_003564102.1
BdHKT9	*Brachypodium distachyon*	Purple false brome	XP_003563514.1
CaHKT1	*Cochlearia anglica*	Scurvy grass	AFH37929.1
DfHKT	*Diplachne fusca*	Brown beetle grass	AEM55592.1
EcHKT1;1	*Eucalyptus camaldulensis*	River redgum	AF176035_1
EcHKT1;2	*Eucalyptus camaldulensis*	River redgum	AF176036_1
EsHKT1	*Eutrema salsugineum*	Saltwater cress	AFJ23835.1
GmHKT1	*Glycine max*	Soybean	XP_003540998.1
HbHKT	*Hordeum brevisubulatum*	Short-awned barley	AER42622.1
HvHKT1;5	*Hordeum vulgare*	Barley	ABK58096.1
HvHKT4	*Hordeum vulgare*	Barley	AEM44690.1
HvHKT2;1	*Hordeum vulgare*	Barley	AEM55590.1
McHKT1;1	*Mesembryanthemum crystallinum*	Ice plant	AF367366_1
McHKT1;2	*Mesembryanthemum crystallinum*	Ice plant	AAO73474.1
MtHKT1;5	*Medicago truncatula*	Alfalfa	AES77170.1
OgHKT1	*Oryza glumipatula*	Wild rice	ABD15858.1
OrHKT1	*Oryza rufipogon*	Red rice	AAY33540.1
OsHKT1;1	*Oryza sativa*	Rice	Q7XPF8.2
OsHKT1;3	*Oryza sativa*	Rice	Q6H501.1
OsHKT1;5	*Oryza sativa*	Rice	A2WNZ9.2
OsHKT2;1	*Oryza sativa*	Rice	A2YGP9.2
OsHKT2;2	*Oryza sativa*	Rice	Q93XI5.1
OsHKT2;3	*Oryza sativa*	Rice	Q8L481.1
OsHKT2;4	*Oryza sativa*	Rice	Q8L4K5.1
PaHKT1	*Phragmites australis*	Common reed	BAE44384.1
PtHKT1	*Populus trichocarpa*	Poplar	EEF03794.1
PutHKT2;1	*Puccinellia tenuiflora*	Alkali grass	ACT21087.1
SbiHKT1;5	*Sorghum bicolor*	Sorghum	EES02856.1
SbiHKT1;3	*Sorghum bicolor*	Sorghum	EES04614.1
SbiHKT2;3	*Sorghum bicolor*	Sorghum	EER90327.1
SbHKT1	*Salicornia bigelovii*	Dwarf saltwort	ADG45565.1
SmHKT1	*Selaginella moellendorffii*	Starry spike moss	EFJ18587.1
SsHKT1	*Suaeda salsa*	Seepweed	AAS20529.2
TaHKT1;5-D	*Triticum aestivum*	Bread wheat	ABG33949.1
TaHKT2;1	*Triticum aestivum*	Bread wheat	AAA52749
TaHKT1;5-B1	*Triticum aestivum*	Bread wheat	ABG33947.1
TaHKT1;5-B2	*Triticum aestivum*	Bread wheat	ABG33948.1
TmHKT1;5-A	*Triticum monococcum*	Einkorn wheat	ABG33946.1
ThHKT1	*Thellungiella halophila*	Salt cress	BAJ34563.1
VvHKT1	*Vitis vinifera*	Grape vine	XP_002270986.1
VvHKT1;3	*Vitis vinifera*	Grape vine	XP_002267717.1
ZmHKT1	*Zea mays*	Maize/corn	AEK27028.1
ScTRK1	*Saccharomyces cerevisiae*	Yeast	AAA34728
ScTRK2	*Saccharomyces cerevisiae*	Yeast	AAA35172
VpTrkH	*Vibrio parahaemolyticus*	Not applicable	Q87TN7.1

**Table 2:** The amino acid residues, given in three-letter codes, lining pores of the EcHKT1;2, OsHKT1;5 and VpTrkH transporters. The predicted selectivity filter residues are underlined.

Protein	Accession number	Pore residues 1							Residue number
EcHKT1;2	AF176036_1	Thr	Thr	Val	Ser	Ser	Met	Ser	91–97
		Ala	Ser	Cys	Gly	Phe	Val	Pro	259–265
		Arg	His	Thr	Gly	Glu	Thr	Val	383–389
		Gly	Asn	Val	Gly	Phe	Thr	Thr	488–494
OsHKT1;5	A2WNZ9.2	Thr	Val	Ser	Ser	Met	Val	Ala	73–79
		Ala	Asn	Cys	Gly	Phe	Val	Pro	261–267
		Arg	His	Ser	Gly	Glu	Met	Val	388–394
		Gly	Asn	Val	Gly	Phe	Ser	Thr	492–498
VpTrkH	Q87TN7.1	Thr	Thr	Thr	Gly	Ala	Thr	Val	110–116
		Ala	Ile	Gly	Gly	Phe	Ser	Thr	219–225
		Thr	Thr	Ala	Gly	Phe	Thr	Thr	319–325
		Asn	Asn	Leu	Gly	Pro	Gly	Leu	436–442

1 Residues are coloured according to conservation predictions analysed by the ConSurf server [81].